# Supplementary material for: Pan-neuroblastoma analysis reveals age- and signature-associated driver alterations
Source: Nat Commun. 2020 Oct 14;11:5183. doi: 10.1038/s41467-020-18987-4 (PMC7560655; doi:10.1038/s41467-020-18987-4)
Supplement: Supplementary file 1 — Supplementary Information [file 41467_2020_18987_MOESM1_ESM.pdf]

Pan-neuroblastoma analysis reveals age- and signature-associated driver alterations

Brady et al.

Supplementary Information

Contents:

Supplementary Tables 1-2

Supplementary Figures 1-21

Supplementary Table 1: Summary of cohort statistics

Stage	# of samples	% of samples
1	131	19.1%
2A	34	5.0%
2B	49	7.2%
3	28	4.1%
4S	31	4.5%
4	412	60.1%
Total*	685	100.0%

*Samples with WGS or WES

Value	Age in years
minimum	0.00 (1 day)
25th percentile	1.15
median	2.73
75th percentile	4.63
maximum	29.20

***MYCN* status**	**# of samples**	**% of samples**
MYCN altered	128	18.7%
MYCN non-altered	557	81.3%
Total*	685	100.0%

*Samples with WGS or WES

Supplementary Table 2: Neural genes significantly decreased in expression in signature 18-positive neuroblastomas

These genes were used as input to ssGSEA to determine a neural score for each sample.
Each had adjusted *P* values less than 0.05 by Limma analysis (comparing signature 18-negative vs. -positive neuroblastomas) and have neural functions per the literature.

DST
GABBR1
PIRT
PAFAH1B1
ZBTB4
PMP22
POMGNT1
DAB1
TTLL7
WDR47
MYO5A
RBFOX1
SCN3A
SCN7A
GLS
TMEFF2
PYGB
GRID2
BTBD9
BAI3
RIMS1
TMEM30A
SERINC1
AGPAT4
PLXNA4
ADCY1

Supplementary Figure 1

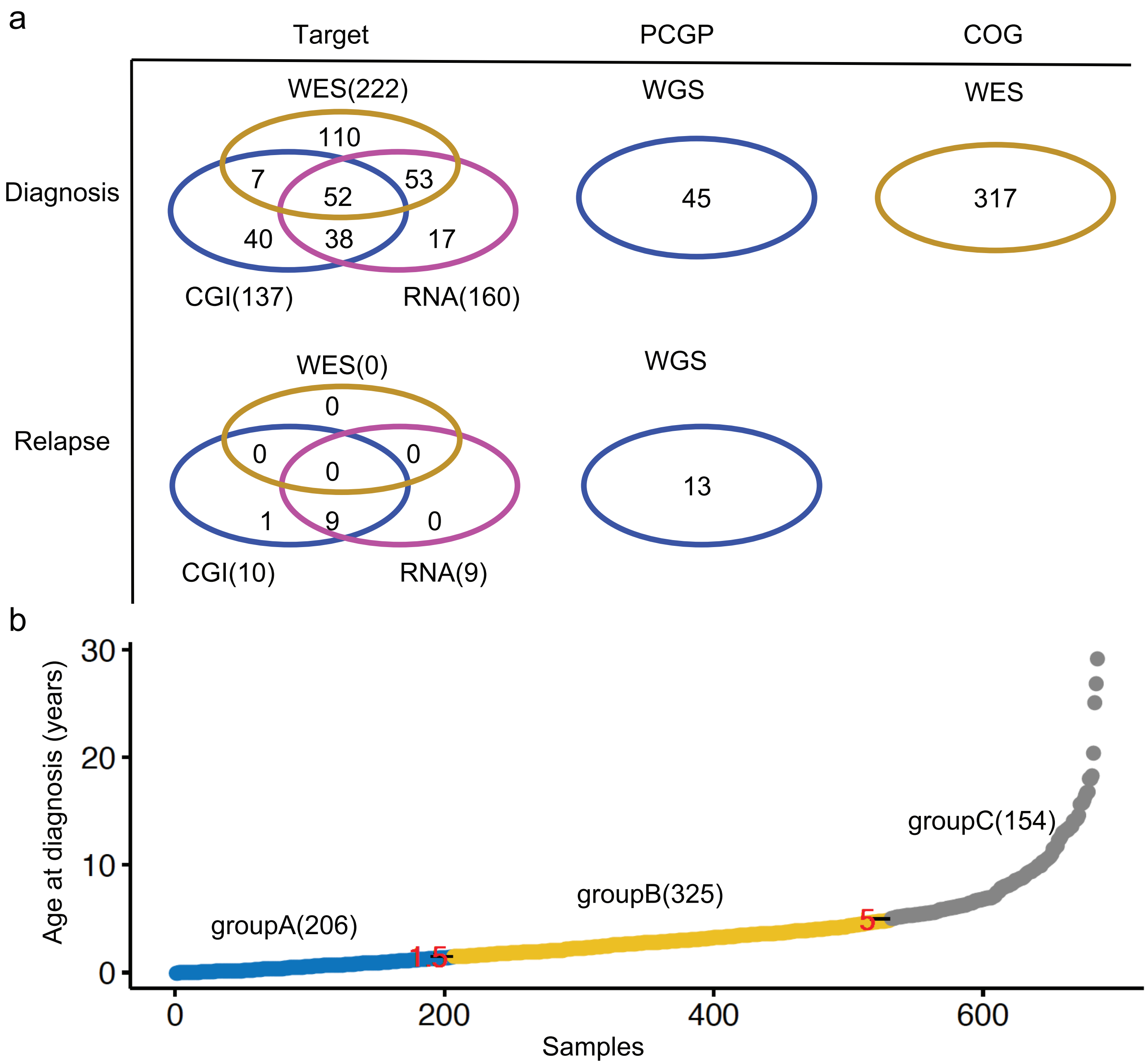

Supplementary Figure 1. Cohort description and age distribution. (**a**) Venn diagrams showing the type of sequencing performed in various samples from the cohort. WES indicates exome sequencing and WGS indicates whole-genome sequencing. CGI indicates Complete Genomics sequencing which is a whole-genome sequencing approach that varies from standard Illumina sequencing. (**b**) Age at diagnosis of 685 samples across the cohort that had WGS or WES, including both diagnosis and relapse samples. Color indicates samples in group A (blue, <1.5 years), group B (yellow, 1.5-5 years), and group C (gray, >5 years).

Supplementary Figure 2

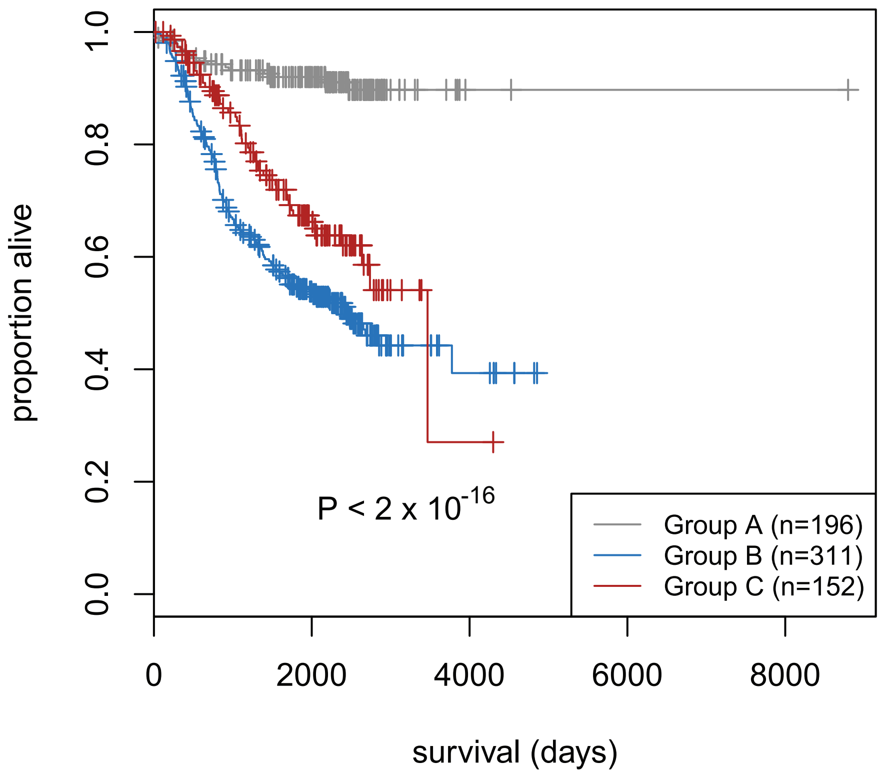

Supplementary Figure 2. Survival rate among age groups. Survival analysis of age groups A, B, and C, including 659 diagnosis samples which had WGS or WES (and which also had survival information). Ticks indicate censoring. *P* value is by two-sided log-rank test.

Supplementary Figure 3

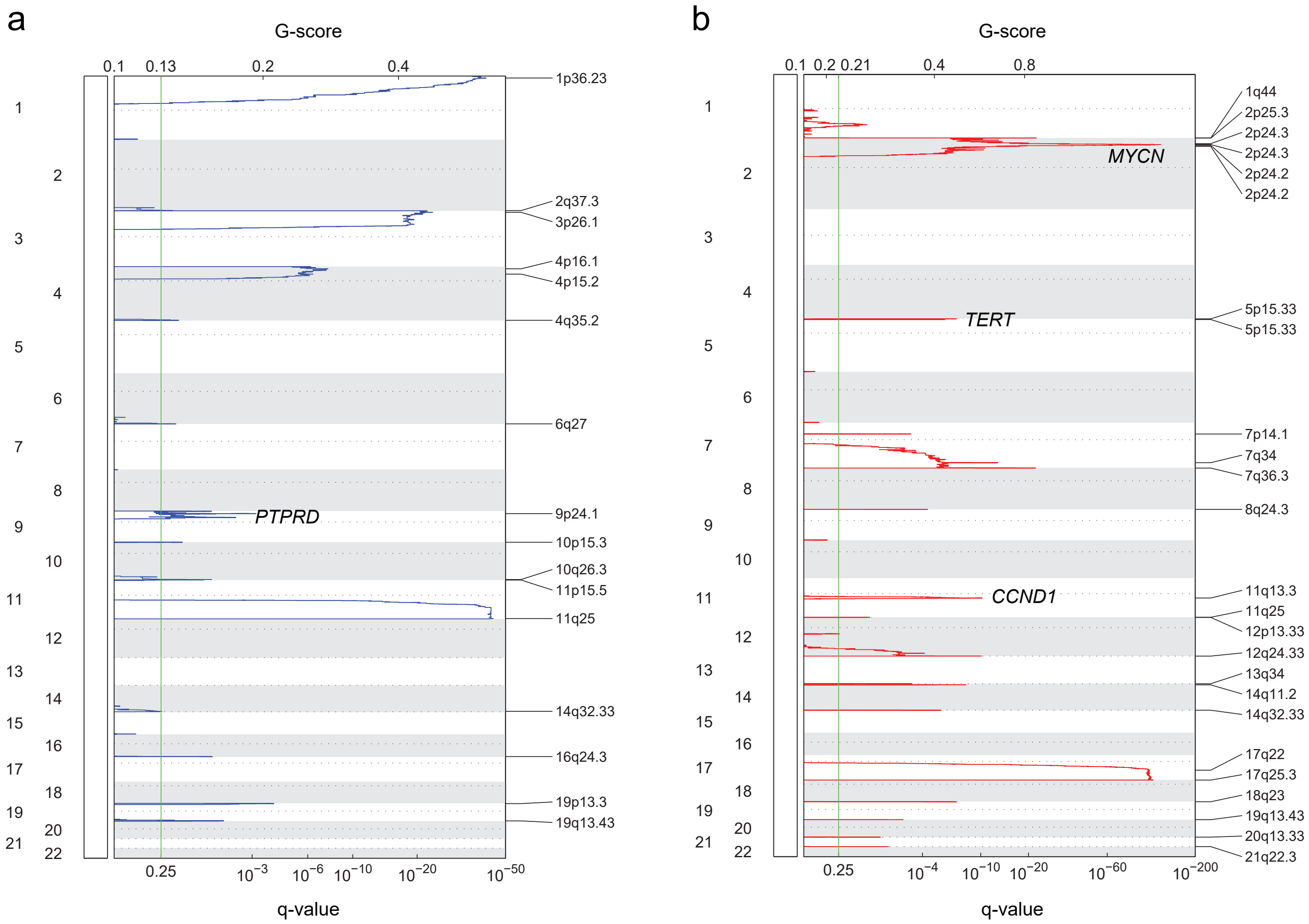

Supplementary Figure 3. Significant copy number alteration regions in 205 neuroblastoma samples. Significant deletion regions and gain regions are shown in (**a**) and (**b**), respectively based on GISTIC 2.0 analysis of 205 samples with WGS, including both diagnosis (n = 182) and relapse (n = 23). G score is indicated on the top x-axis and q-value on bottom x-axis. Y-axis represents genomic position from chromosome 1 to 22. Specific genes altered in chromosomal regions are indicated by nearby text.

Supplementary Figure 4

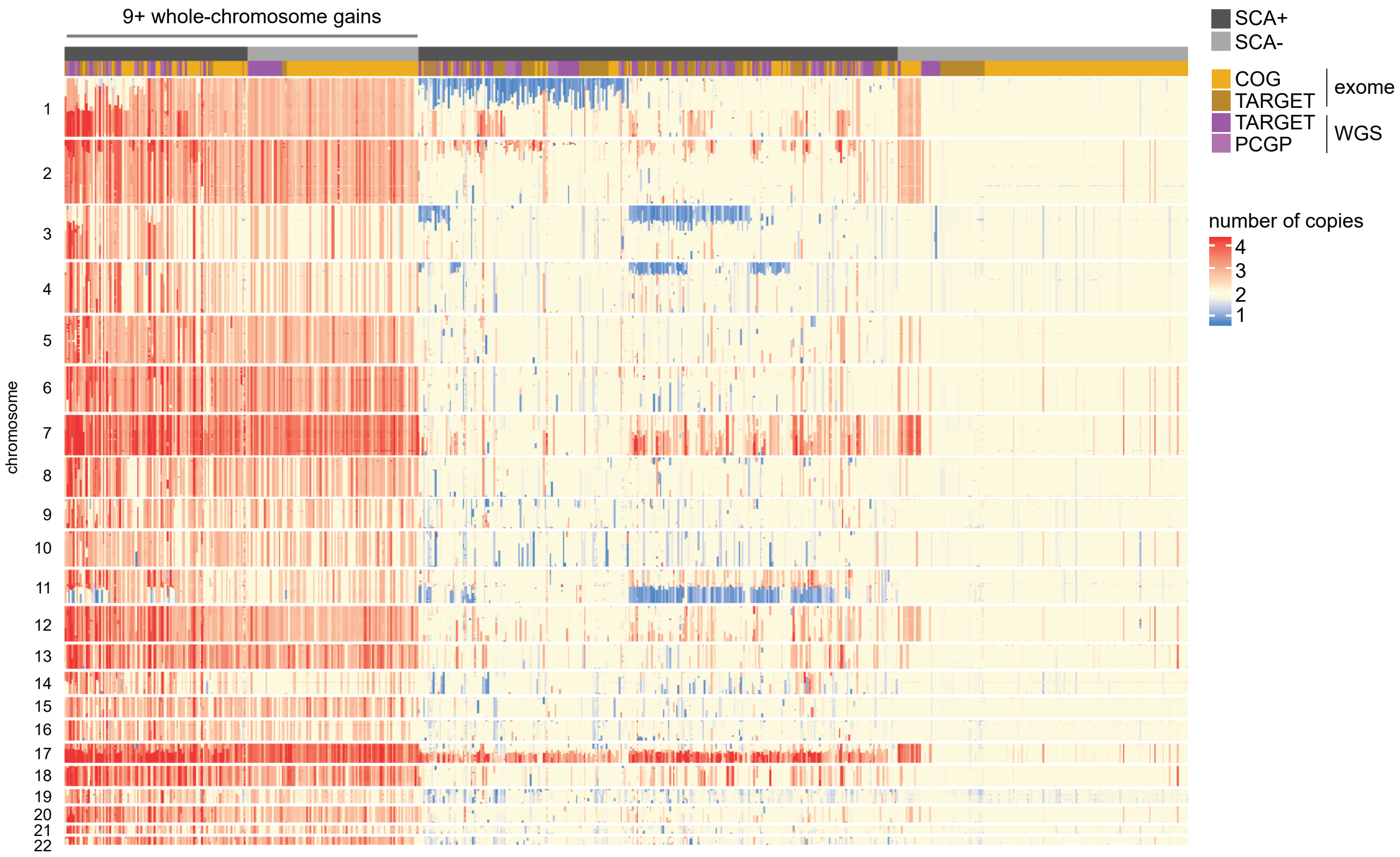

Supplementary Figure 4. Copy number profiles across the cohort. Copy level, indicated by linear copy (e.g. 2 indicates diploid), is shown for each of 685 samples (columns) sequenced by WGS or WES including both diagnosis (n = 662) and relapse (n = 23) samples. Chromosomes and positions are sorted from top to bottom. Red indicates above-diploid copy (gain), while blue indicates below-diploid (loss). The type of sequencing and source cohort is indicated at the top in shades of purple or gold-brown. Samples with segmental chromosome copy alterations (SCA+) in any chromosome are indicated in dark-gray at top, while samples lacking such alterations (SCA-) are shown in light-gray; samples with 9 or more whole-chromosome gains are also indicated on the left (see text at top-left). Source data are provided as a Source Data file.

Supplementary Figure 5

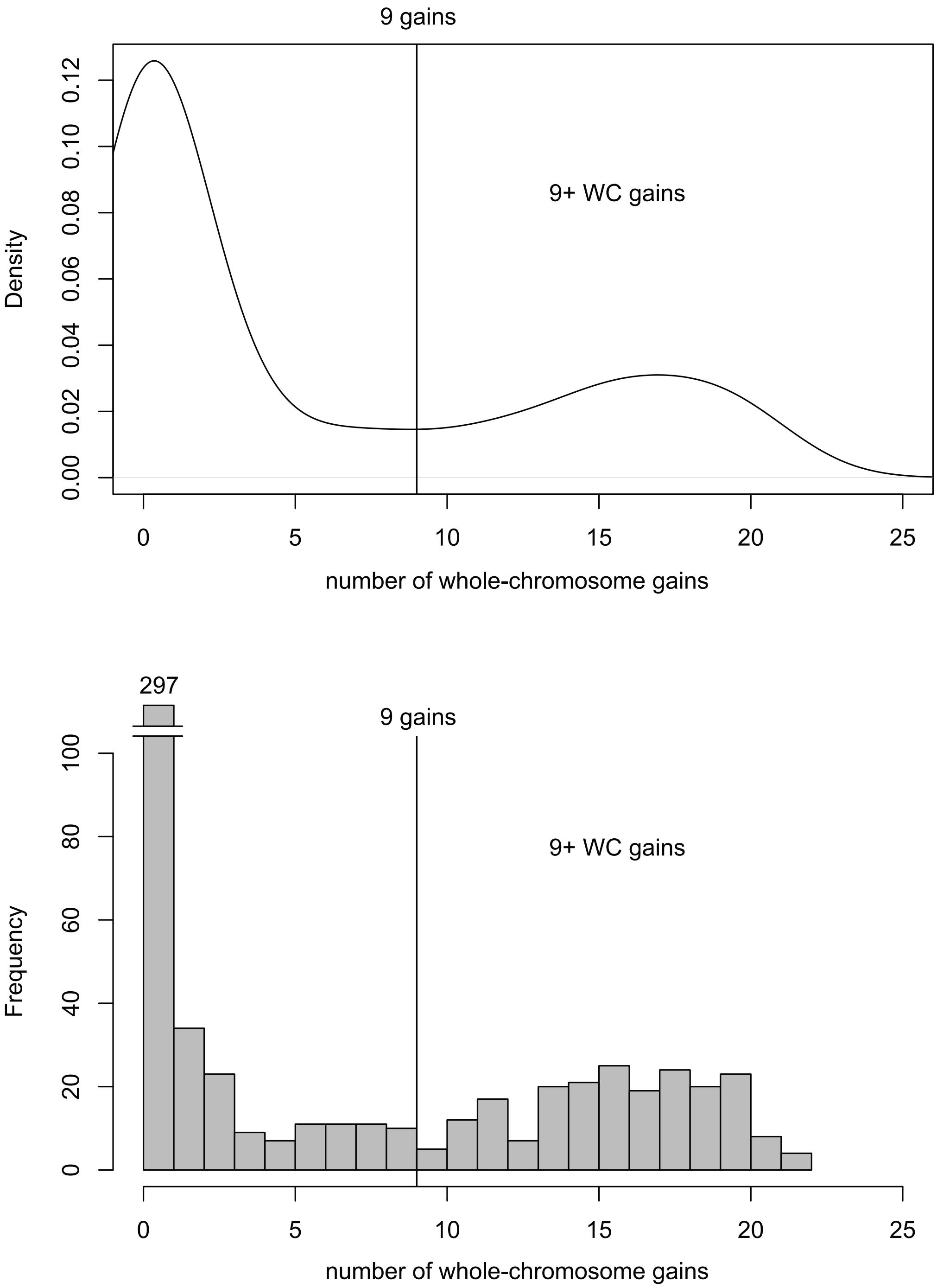

Supplementary Figure 5. Identification of a natural cutoff for whole-chromosome gains. The number of chromosomes with whole-chromosome gains (x-axis) was determined for each of 685 samples with WGS or WES, and density plot analysis was performed (top) or histogram analysis (bottom). Diagnosis (n = 662) and relapse (n = 23) samples were included. 9 gains was the local minimum in density plot analysis and thus samples with 9 or more gains were considered part of the "9+ WC gains" group. Source data are provided as a Source Data file.

Supplementary Figure 6

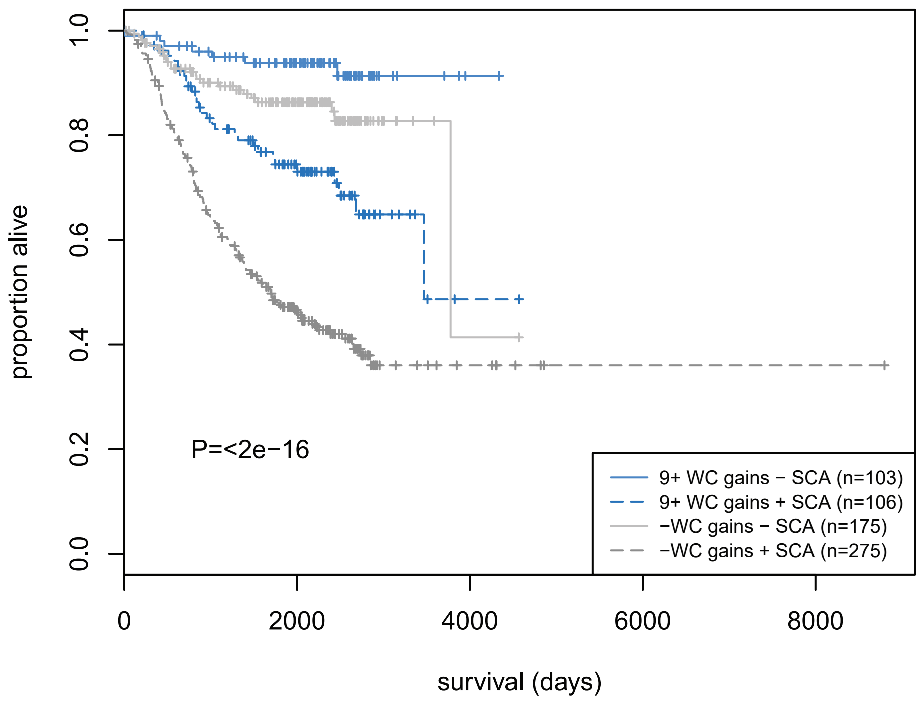

Supplementary Figure 6. Survival by presence of 9 or more whole-chromosome gains and segmental chromosome copy alterations. Survival curve showing survival of samples with 9 or more whole-chromosome gains (blue, "9+ WC gains") vs. zero to 8 whole-chromosome gains (gray, "-WC gains") patients. Patients are also split based on presence of any segmental chromosome copy alterations in any chromosome ("SCA"; a dotted line indicates an SCA is present, while solid line indicates absence). The number of samples in each group is indicated in parentheses. Only diagnosis samples were included (n = 659 diagnosis samples with WGS or WES, and which also had survival information available). *P* value is by two-sided log-rank test. Source data are provided as a Source Data file.

Supplementary Figure 7

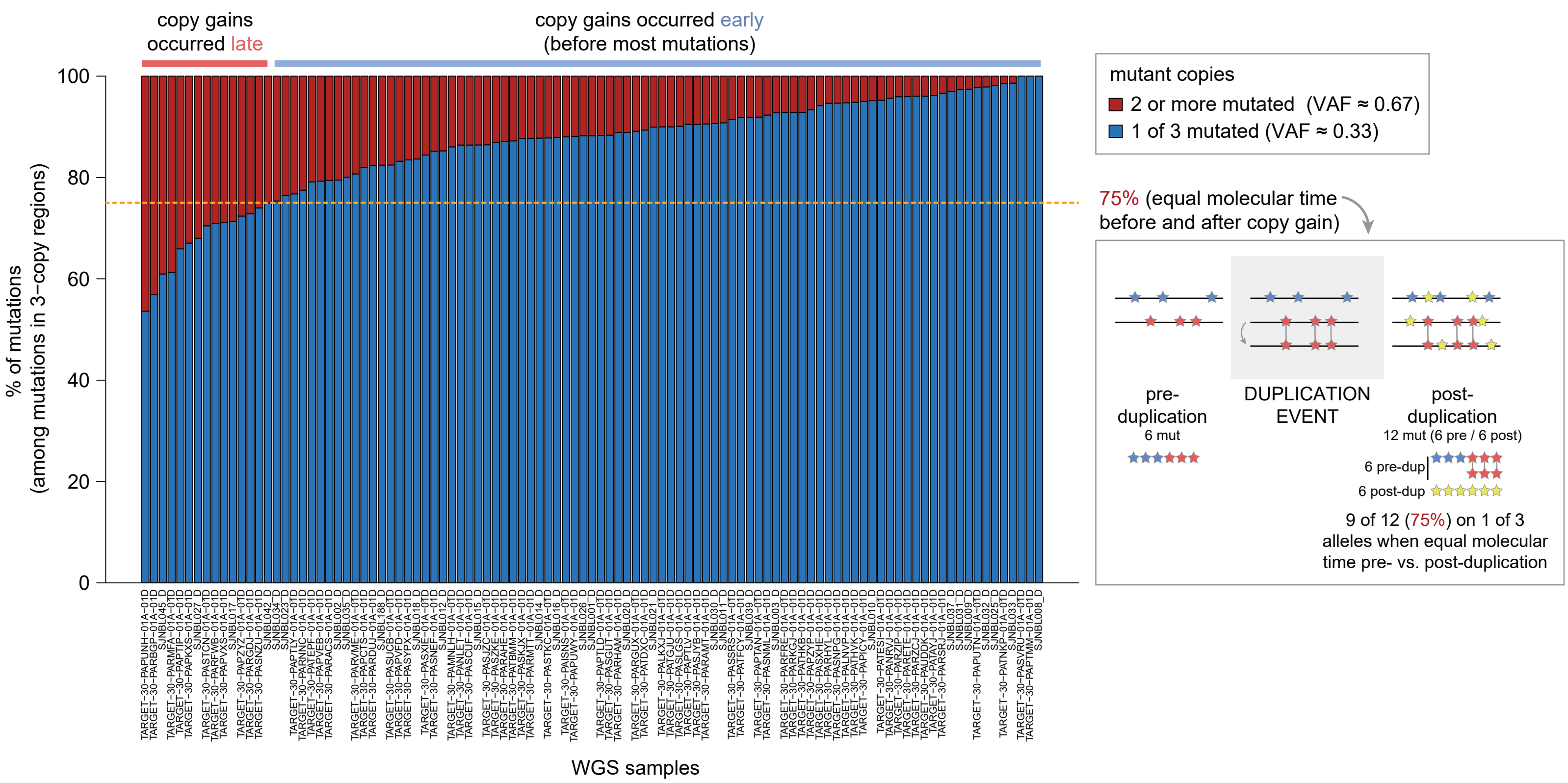

Supplementary Figure 7. Copy gains occur prior to the majority of point mutation in most neuroblastomas. Bar plot representing the percentage of mutations in 3-copy regions (y-axis) where 1 of 3 alleles was mutated (blue) or 2 or more out of 3 alleles was mutated (red; these are early mutations that occur prior to the copy gain) using 103 out of the 182 diagnosis WGS samples (x-axis) with at least 70% tumor purity and at least 20 mutations in 3-copy regions. The molecular timepoint where an equal number of mutations occurred before and after the copy gains is indicated by a yellow dotted line (75%). A justification for 75% being the timepoint of interest is shown to the right. An arbitrary chromosome is shown as two horizontal black lines representing the two copies of the chromosome with 6 somatic mutations, and somatic mutations on one copy are shown as 3 blue stars and on the other copy as 3 red stars. Once one of the chromosomes undergoes duplication, 50% of the mutations are on 1 of 3 copies and 50% on 2 of 3 copies. After 6 additional mutations (equal mutation time before and after copy changes), which all happen on 1 of 3 copies, 9 of 12 mutations (75%) occur on 1 of 3 copies. Samples with more than 75% of variants in 1 of 3 alleles (horizontal thick blue bar; 85% of samples) had early copy gains where most mutations occurred after the copy gains (see Methods for justification). Source data are provided as a Source Data File.

Supplementary Figure 8

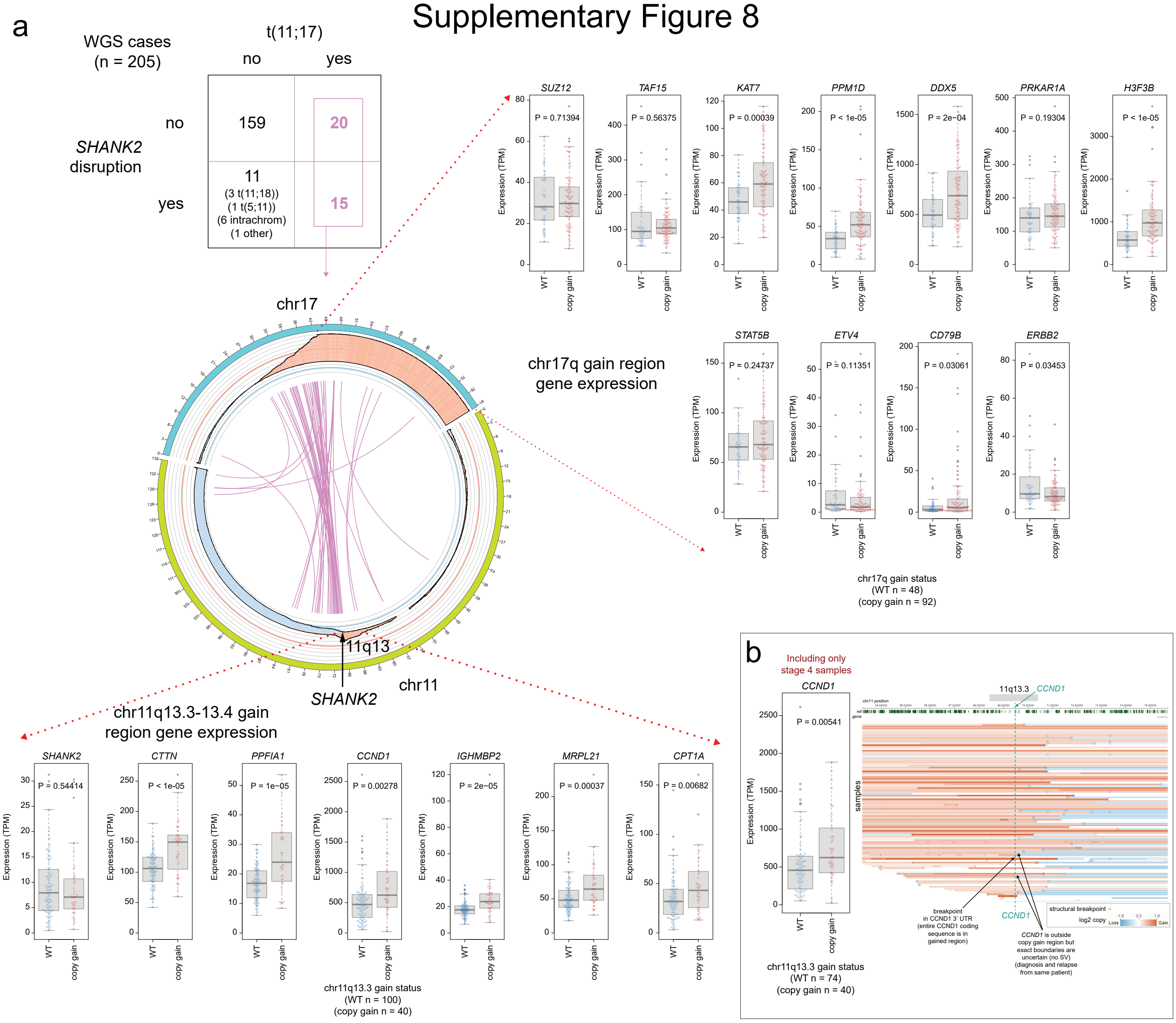

Supplementary Figure 8. *SHANK2* alterations and t(11;17) translocations. (**a**) Top-left shows the number of samples with t(11;17), *SHANK2* disruption, or both, among 205 diagnosis or relapse samples with WGS. t(11;17) translocations frequently disrupted the *SHANK2* gene on chromosome 11 (15 cases) but also occurred at other non-*SHANK2* (but nearby) loci on chromosome 11 (20 cases). Circos plot shows structural alterations between chromosomes 11 and 17 frequently disrupting *SHANK2* on chromosome 11 (n = 35 samples are shown; 15 of these disrupt *SHANK2*). Red indicates copy gain, blue indicates copy loss, and copy data represent the mean copy number in the 35 patients with t(11;17) translocations. Purple lines indicate inter-chromosomal translocations and each line represents one variant in a specific patient. Expression data to the right show boxplots of gene expression for genes in chromosome 17 gain regions, which were associated with t(11;17) translocations. Box represents interquartile range (25th to 75th percentile); middle bar represents median. Whiskers are as described in the R boxplot function documentation (a 1.5*interquartile range rule is used). Genes shown include Sanger Cancer Gene Census genes in the gained region annotated as oncogenes gained in solid tumors. *P* values are by two-sided Wilcoxon rank-sum test, and samples are grouped into 17q-gained samples (copy gain), and non-gained (WT); these copy gains are frequently associated with t(11;17). At the bottom, gene expression data are shown for selected genes in 11q13.3-11q13.4, by 11q13.3 gain status. t(11;17) often occurs at one terminus of 11q13 gains. Gene expression boxplots include 140 diagnosis samples with both RNA-Seq and DNA sequencing (WGS or WES). (**b**) Left, comparison of *CCND1* expression between 11q13.3-gained or -WT samples including only 114 stage 4 samples, to show that the *CCND1* increase in panel (a) is not confounded by stage. *P* value is by two-sided Wilcoxon rank-sum test. Right, copy status of samples with copy alterations in or around 11q13, to show that most such gains include *CCND1* (copy gained regions are shown in red, deleted regions in blue, and *CCND1*'s location is shown as a turquoise dotted line; samples are shown as rows, and the x-direction represents chromosome 11 coordinates).

Supplementary Figure 9

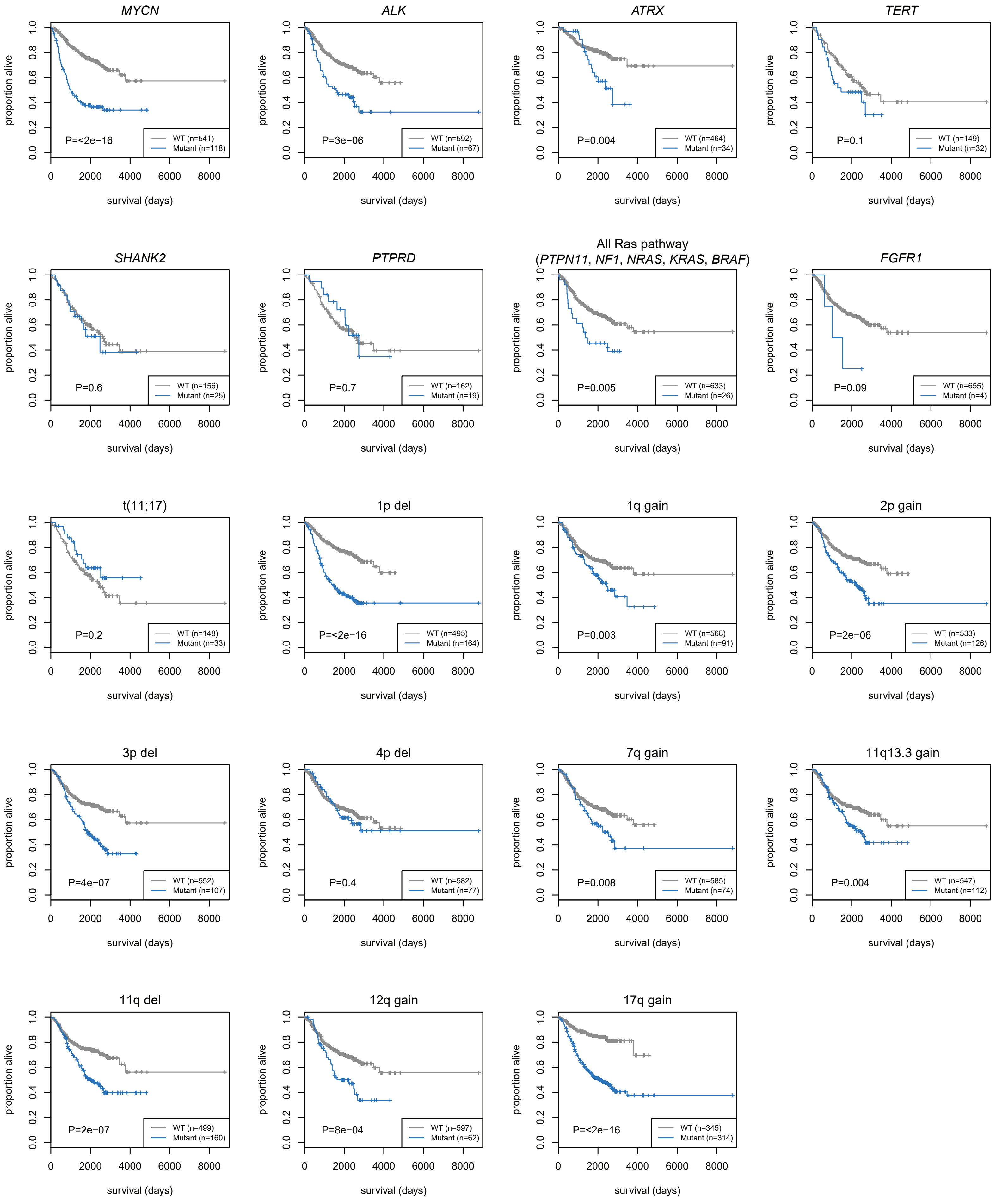

Supplementary Figure 9. Survival rates of recurrent aberrations. Survival analysis of indicated driver alterations; samples with indicated genes or chromosomal regions altered/mutated (Mutant) vs. wild-type (WT) are compared. *P* values are by two-sided log-rank test. "Del" indicates segmental copy loss and "gain" indicates segmental copy gain. Ticks represent censoring. Only diagnosis and not relapse samples were analyzed. For most aberrations, 659 diagnosis samples with WGS or WES (and available survival information) were analyzed, but 181 samples with WGS were analyzed for *PTRPD*, *SHANK2*, t(11;17), and *TERT* (since these are largely affected by SVs), and 498 for *ATRX* (including WGS samples plus samples with WES combined with targeted sequencing of the entire *ATRX* gene, which could detect both *ATRX* SVs and sequence mutations). For the All Ras Pathway plot, Mutant samples include those with genetic alterations in any of the Ras pathway genes indicated, and WT refers to samples with none of them altered.

Supplementary Figure 10

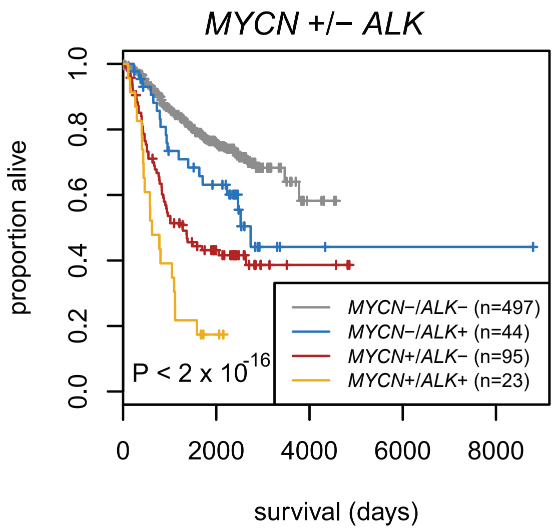

Supplementary Figure 10. Survival rates by *MYCN* and *ALK* status. Log-rank survival analysis of samples with *MYCN* alterations only, *ALK* alterations only, both, or neither (n = 659 diagnosis samples with WGS or WES, plus survival information).

Supplementary Figure 11

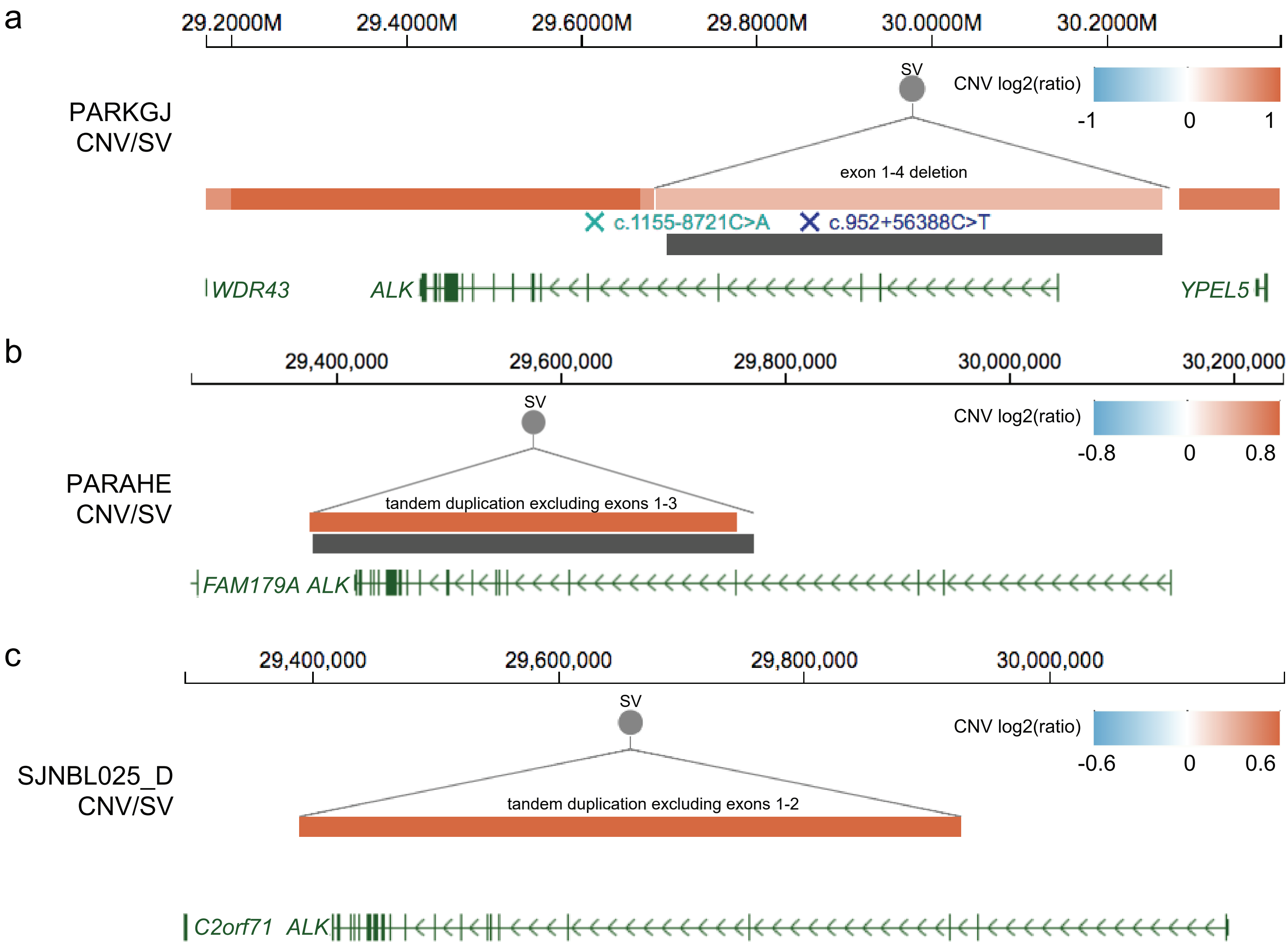

Supplementary Figure 11. Focal deletion and focal tandem duplications in *ALK*. (**a**) Exon 1 to exon 4 deletion in *ALK* in PARKGJ. Copy gains are indicated by red color as in legend, and structural alterations are shown as lines connecting the joined regions. The structure of the *ALK* gene is shown below. (**b** and **c**) Focal tandem duplication of *ALK* without first 3 exons and first 2 exons in PARAHE and SJNBL025, respectively.

Supplementary Figure 12

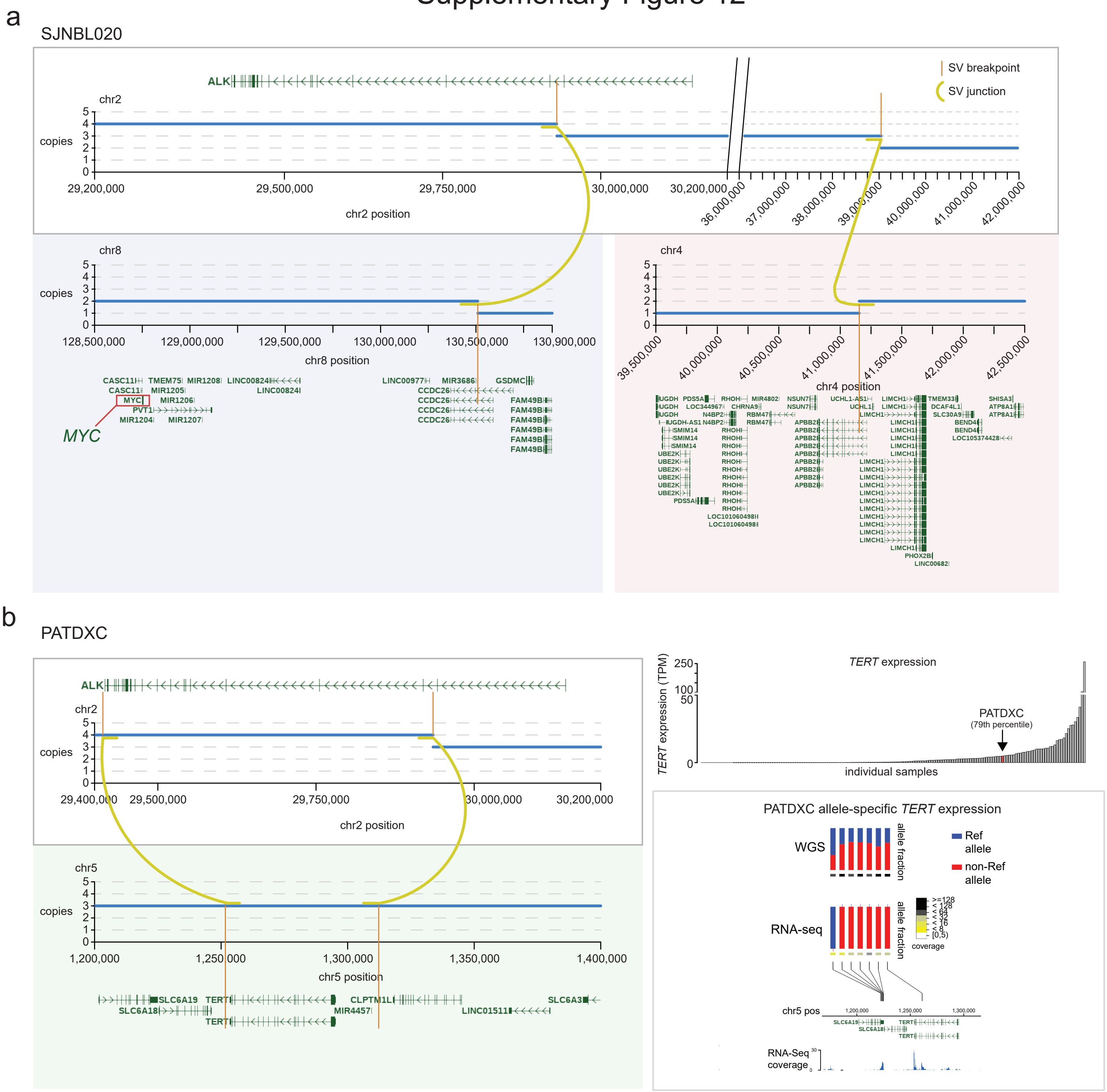

Supplementary Figure 12. Chromosomal translocations of *ALK* in SJNBL020 and PATDXC. (**a**) *ALK* translocation in SJNBL020 bringing intron 2 of *ALK* near the *MYC* locus on chromosome 8. Copy number is indicated along y-axes for indicated chromosomes, rounded to the nearest integer, which gene annotations above or below graphs. SV breakpoints are shown in orange, with inter-chromosomal junctions shown in yellow. (**b**) *ALK* translocation in PATDXC joining intron 2 of *ALK* to the *TERT* locus on chromosome 5. Top-right, *TERT* gene expression by RNA-seq across the TARGET cohort, with PATDXC expression indicated by an arrow. Bottom-right, allele-specific expression of a single allele in the *TERT* region. RefGene gene locations on chromosome 5 are shown at bottom along with RNA-Seq coverage in sample PATDXC. WGS allele fractions for germline SNPs (locations indicated by black lines) detected by WGS in the tumor sample are shown at top, with the reference allele shown in blue and an alternate allele in red; each SNP had both alleles represented by WGS (germline heterozygous variants). However, in RNA-Seq data (middle) only one allele was expressed, as only the reference or only the alternate allele were detected. This is consistent with *cis*-activation of a single allele, consistent with a nearby translocation potentially driving gene expression. Note also that below each allele fraction bar, the sequencing coverage is indicated by yellow-black colors. This event may not have affected *ALK* expression, as only weak allele-specific expression was observed near *ALK*, and *ALK* is expressed at basal levels in neuroblastoma. Source data are provided as a Source Data file.

Supplementary Figure 13

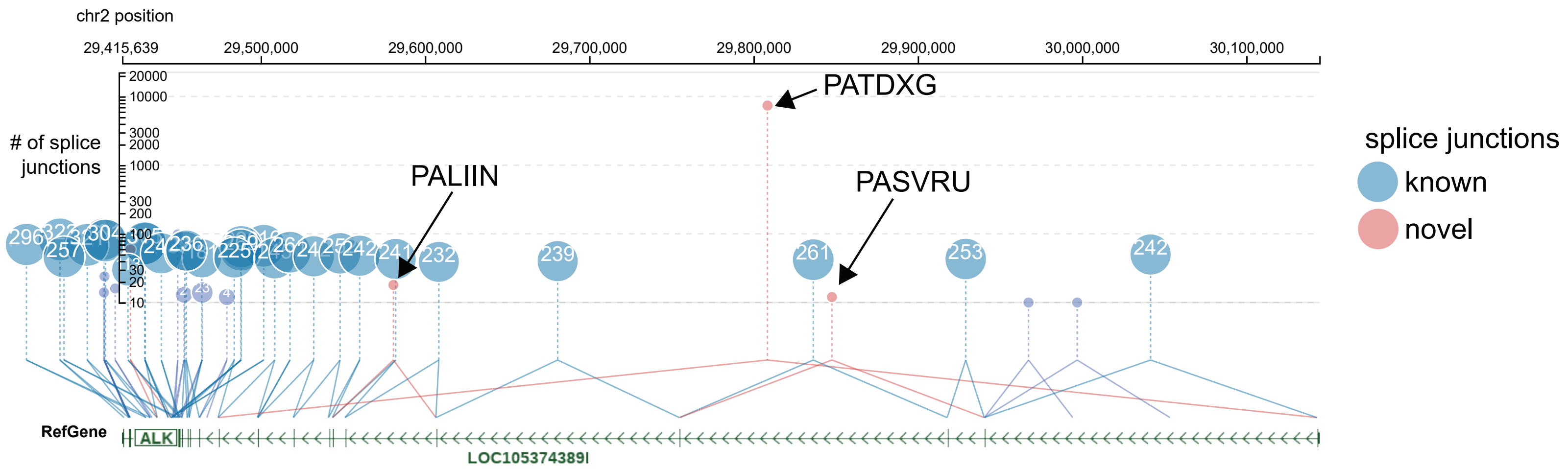

Supplementary Figure 13. *ALK* splicing alterations. Alternative splicing in 3 neuroblastoma samples (PATDXG, PALIIN, and PASVRU). These cases lacked WGS data, and thus it cannot be determined whether these splice changes result from intragenic deletions. In PATDXG, *ALK* was amplified and also had an RNA splice variant joining exon 1 to exon 12 in frame. In PASVRU and PALIIN, we observed exon skipping in 12% and 20% of *ALK* transcripts, respectively, suggesting in-frame deletions of exon 3 or exon 6, respectively. *ALK* transcript structure is shown at bottom, and junction reads from RNA-seq are shown as blue lines (known junctions) or red lines (novel junctions) from RNA-seq. This exon skipping in *ALK* was not observed with high confidence in any other tumor types based on analysis of 353 PCGP cancers consisting of brain tumors (n = 145), solid tumors (n = 199), and hematopoietic cancers (n = 8). Numbers in blue circles indicate the total number of splicing alterations observed across these 353 cancers. >5 reads and >10% ratio were used as novel junction cutoffs.

Supplementary Figure 14

a

MYCN-TERT co-occurrence cases

Sample	*MYCN*	*TERT*
SJNBL023_D	amp	translocation
SJNBL027_D	amp	downstream copy gain
PARNTS	amp	copy gain
PAPUTN	amp/*TERT* translocation	amp/*MYCN* translocation
PARSRJ	amp/*TERT* translocation	downstream amp/*MYCN* translocation

b PAPUTN

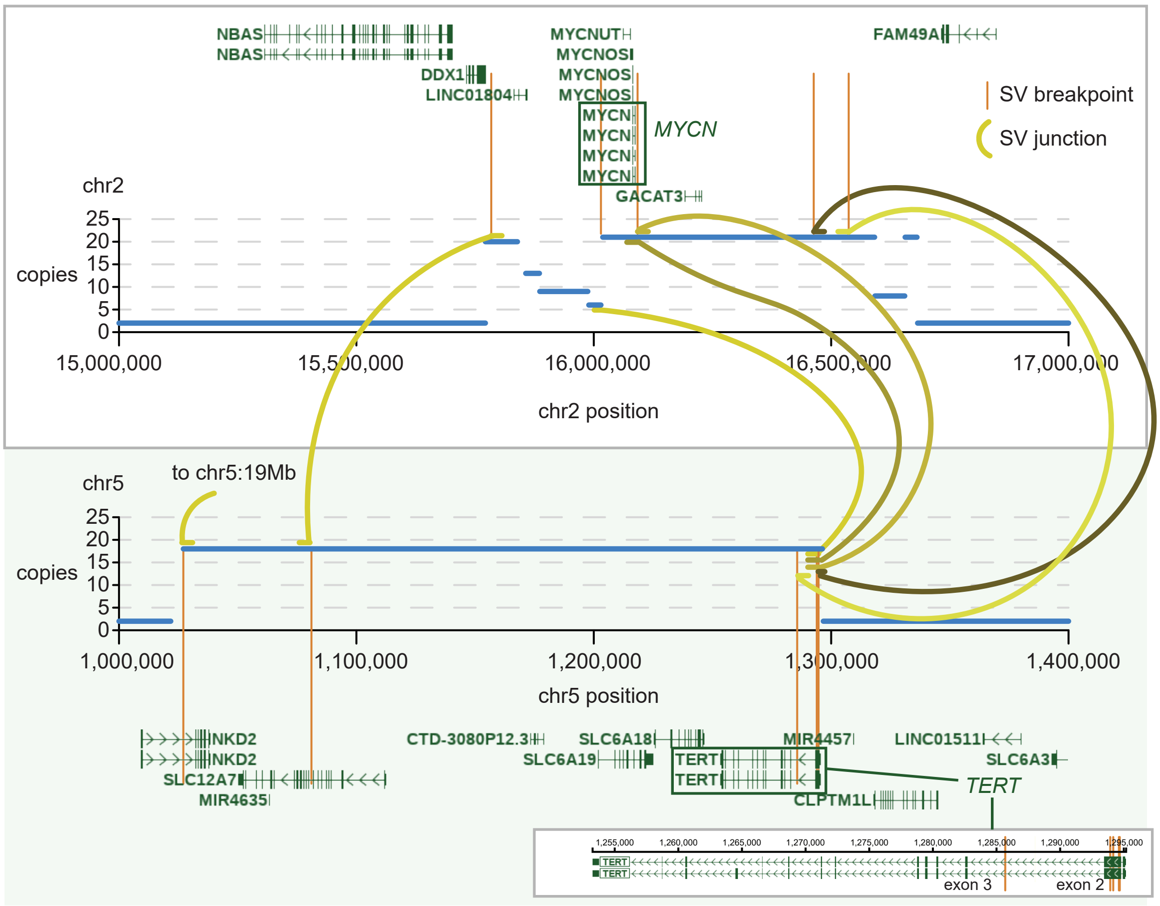

c PARSRJ

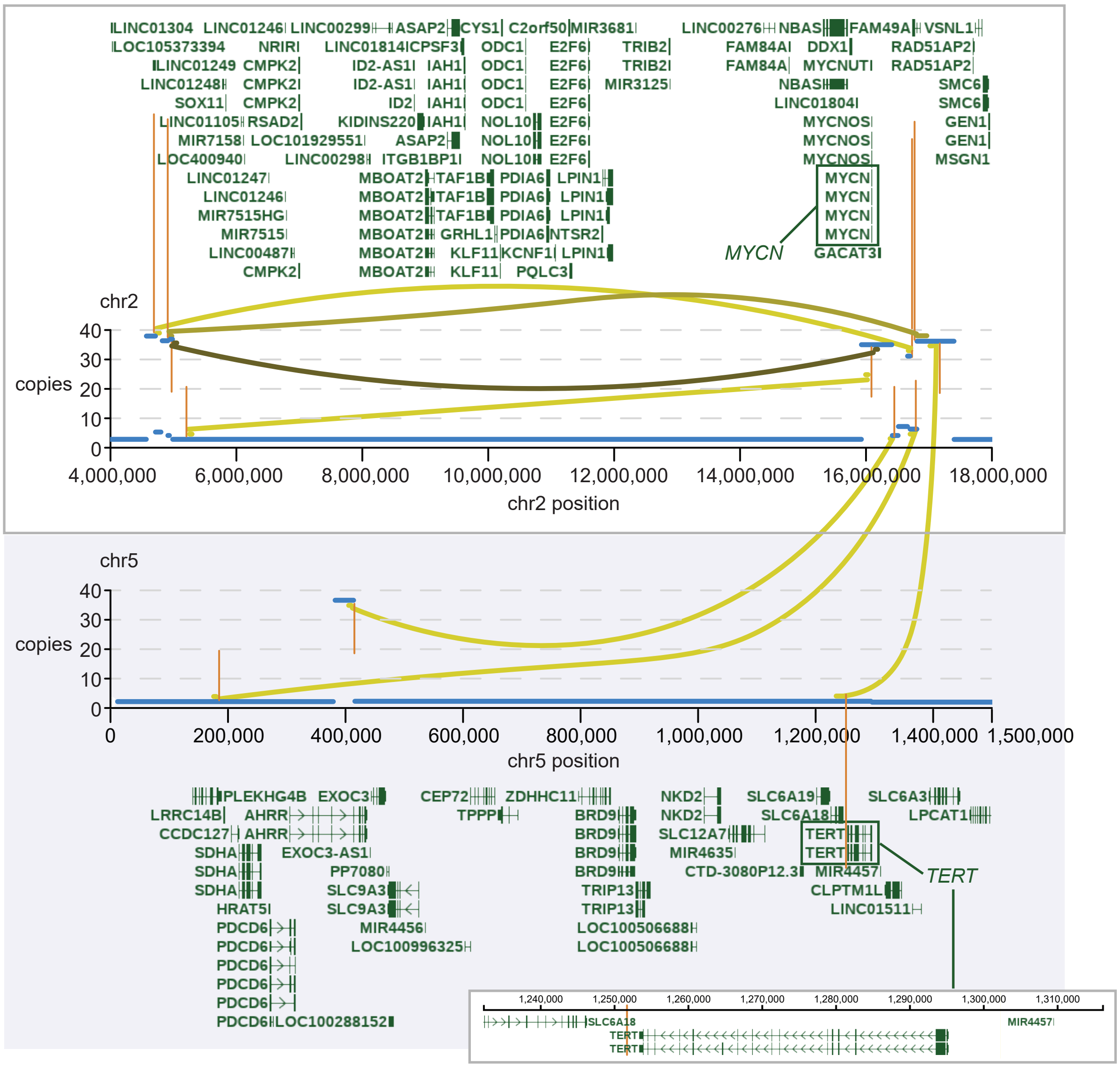

Supplementary Figure 14. Five samples with co-mutation of *MYCN* and *TERT*. (**a**) Five of 205 samples with WGS had alterations in both *MYCN* and *TERT*, as indicated in this table. The type of alteration in each sample is indicated (amp indicates amplification). (**b** and **c**) *MYCN-TERT* region translocations (t(2;5)) in two of the five samples with co-occurrence of *MYCN* and *TERT* alterations. Figures were constructed as in Supplementary Fig. 12. *TERT* insets at bottom of each panel show the location of SV breakpoints in greater detail in relation to *TERT* (intronic in (**b**), outside the gene body in (**c**)).

Supplementary Figure 15

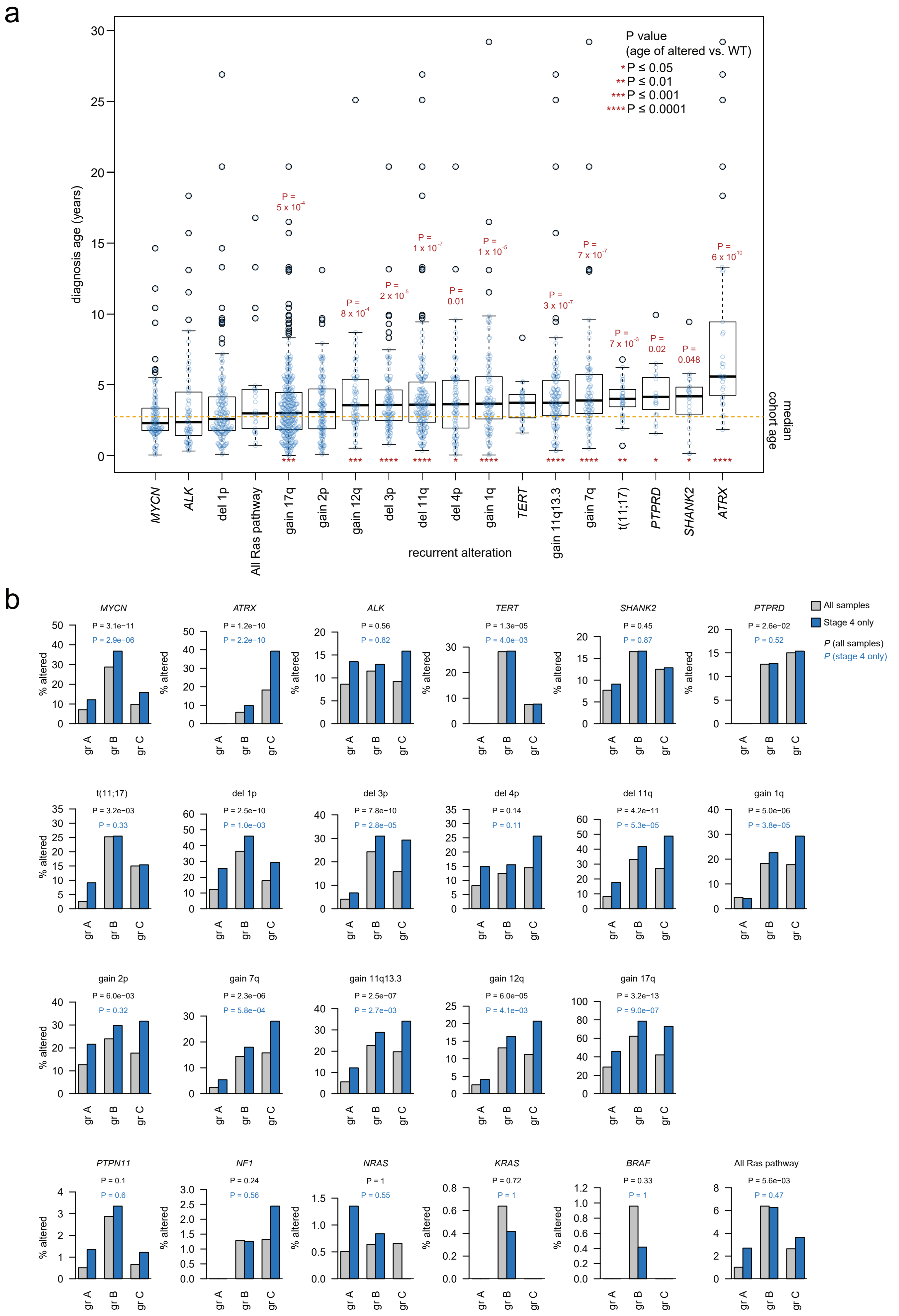

Supplementary Figure 15. Age distributions of recurrent alterations. (**a**) Boxplots of patient ages based on genomic aberration status (including SNVs, indels, CNVs, and SVs); blue points represent individual patients; outliers have additional black outline. We analyzed 662 diagnosis samples with WGS or WES for most variants, except for *TERT*, *PTPRD*, *SHANK2*, and t(11;17) (n = 182 diagnosis WGS samples, as these are primarily SVs), and *ATRX* (n = 499, including WGS samples plus samples with WES combined with targeted sequencing of the entire *ATRX* gene). Box, interquartile range (25th to 75th percentile); middle bar, median. Whiskers are described in R boxplot documentation (a 1.5*interquartile range rule is used). Significance level *, **, *** and **** represent two-sided Wilcoxon rank-sum $P < 0.05$, 0.01, 0.001, and 0.0001, respectively. *P* values were calculated by comparing, for each alteration, the ages of all patients with vs. without the variant. Yellow dotted line, median age for all patients. Numbers of patients with each alteration are reported in Supplementary Data 1. (**b**) Prevalence of each alteration in the three age groups; diagnosis samples were analyzed. Gray bars and black text, analysis of all samples; blue bars and text, stage 4 samples only. *P* values are by two-sided Fisher's exact test. Sample numbers for all-sample comparisons included, for most alterations, 662 diagnosis samples split into 197 (group A), 313 (B), and 152 (C); for *TERT*, *PTPRD*, *SHANK2*, and t(11;17), there were 182 samples split into 39 (A), 103 (B), and 40 (C); and 499 samples for *ATRX*, split into 197 (A), 176 (B), and 126 (C). For stage 4 analysis, for most variants there were 395 diagnosis samples split into 74 (A), 239 (B), and 82 (C); for *TERT*, *PTPRD*, *SHANK2*, and t(11;17), there were 152 samples split into 11 (A), 102 (B), and 39 (C); and 232 samples for *ATRX*, split into 74 (A), 102 (B), and 56 (C). All Ras pathway includes alterations in *PTPN11*, *NF1*, *NRAS*, *KRAS*, or *BRAF*; gr means age group. Deletions and gains refer to segmental chromosome copy alterations (excludes whole-chromosome alterations). Source data are provided in Source Data file.

Supplementary Figure 16

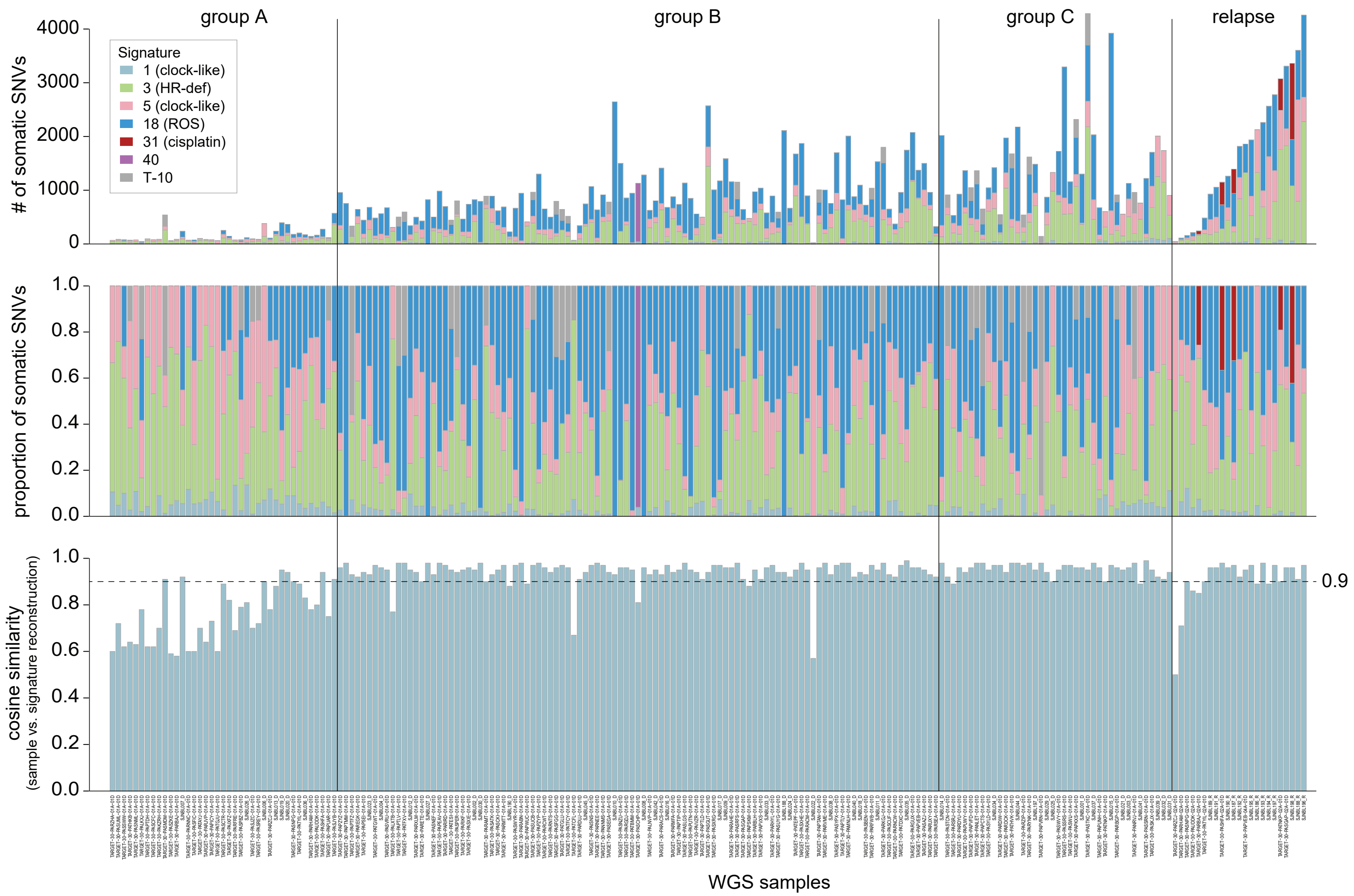

Supplementary Figure 16. Mutational signatures in individual samples. Mutational signatures detected in WGS samples at diagnosis (left; group A, group B, or group C, based on the age groups) or relapse (right); n = 205 samples total. Samples at diagnosis are sorted by age increasing from left to right, while relapse samples are sorted by mutation burden. In the top panel, the number of SNVs caused by each signature is indicated on the y-axis, and color indicates the signature(s) causing mutations in the sample. The middle panel shows the same data as in the top panel, except shown as a proportion of all mutations, rather than absolute mutation numbers. The bottom panel shows the cosine similarity of the sample's mutation spectrum (based on trinucleotide context) and the sample's mathematical reconstruction using mutational signatures, with 0.9 shown as a dotted line as a general guide to indicate samples with the most reliable mutational signatures. Samples with lower mutation burden tended to have lower cosine scores due to greater noise when mutation numbers are small.

Supplementary Figure 17

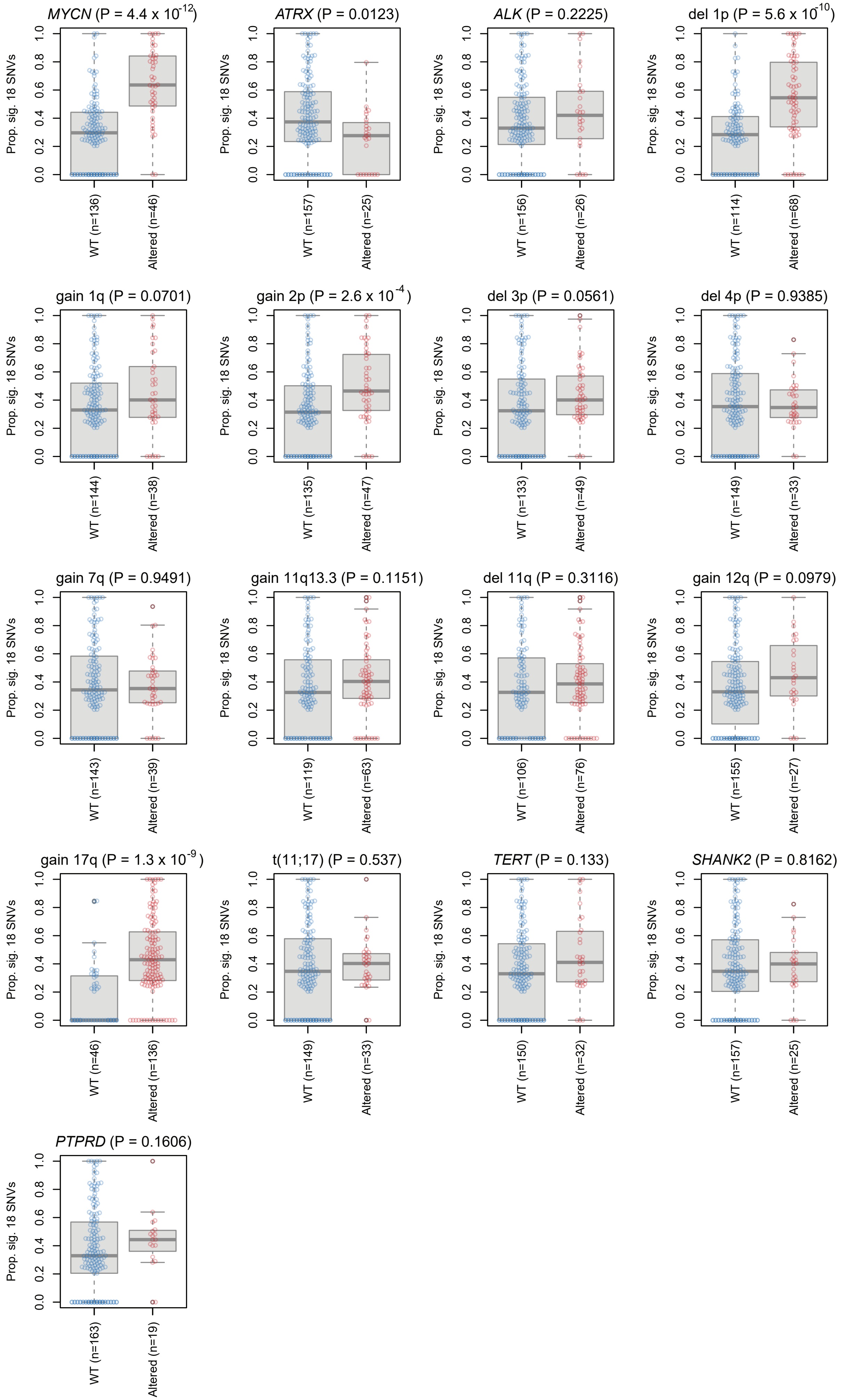

Supplementary Figure 17. Signature 18 correlation with various genetic alterations. Boxplots showing the proportion of SNVs caused by signature 18 in each sample (y-axis) in samples with wild-type (WT) or altered versions of each indicated gene or aberration, with the number of samples in each category indicated by text. 182 diagnosis samples with WGS were analyzed. Box represents interquartile range (from 25th to 75th percentile) and thick bar represents median. Whiskers and outliers are as described in the R boxplot function documentation. (For whiskers, a 1.5*interquartile range rule is used, and outliers are points outside of the whiskers.) Each blue or red point represents one sample. *P* values are by two-sided Wilcoxon rank-sum test. We required a significance level of $\alpha = 2.94 \times 10^{-3}$ using a Bonferroni adjustment for multiple hypothesis testing of the 17 alterations shown. Gains and deletions (del) refer to segmental chromosome copy alterations and exclude whole-chromosome gains.

Supplementary Figure 18

stage 4 samples only

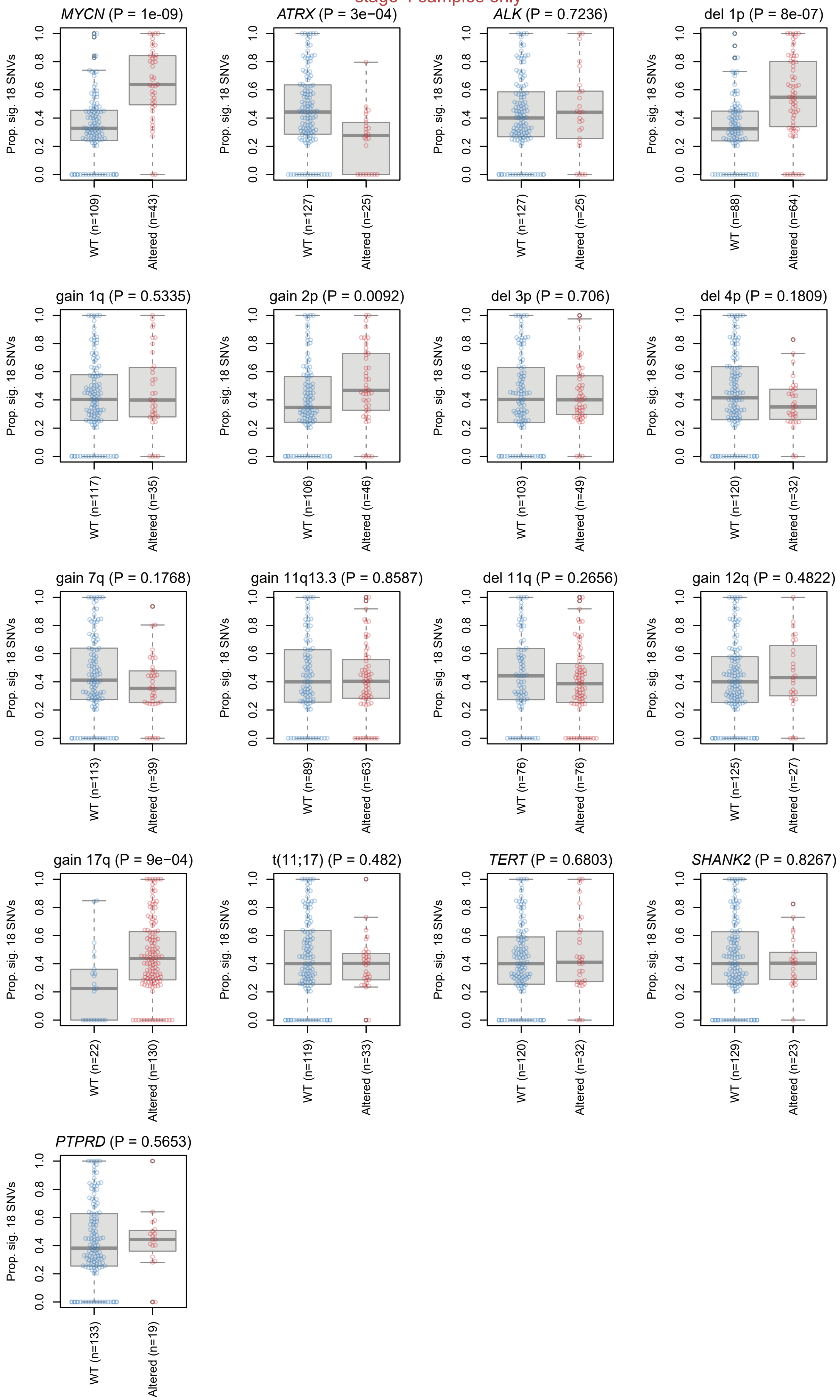

Supplementary Figure 18. Signature 18 correlation with various genetic alterations including only stage 4 samples. Boxplots showing the proportion of SNVs caused by signature 18 in each stage 4 sample (y-axis) in samples with wild-type (WT) or altered versions of each indicated gene or aberration, with the number of samples in each category indicated by text. 152 diagnosis stage 4 samples with WGS were analyzed. Box represents interquartile range (from 25th to 75th percentile) and thick bar represents median. Whiskers and outliers are as described in the R boxplot function documentation. (For whiskers, a 1.5*interquartile range rule is used, and outliers are points outside of the whiskers.) Each blue or red point represents one sample. *P* values are by two-sided Wilcoxon rank-sum test. We required a significance level of $\alpha = 2.94 \times 10^{-3}$ using a Bonferroni adjustment for multiple hypothesis testing of the 17 alterations shown. Gains and deletions (del) refer to segmental chromosome copy alterations and exclude whole-chromosome gains.

Supplementary Figure 19

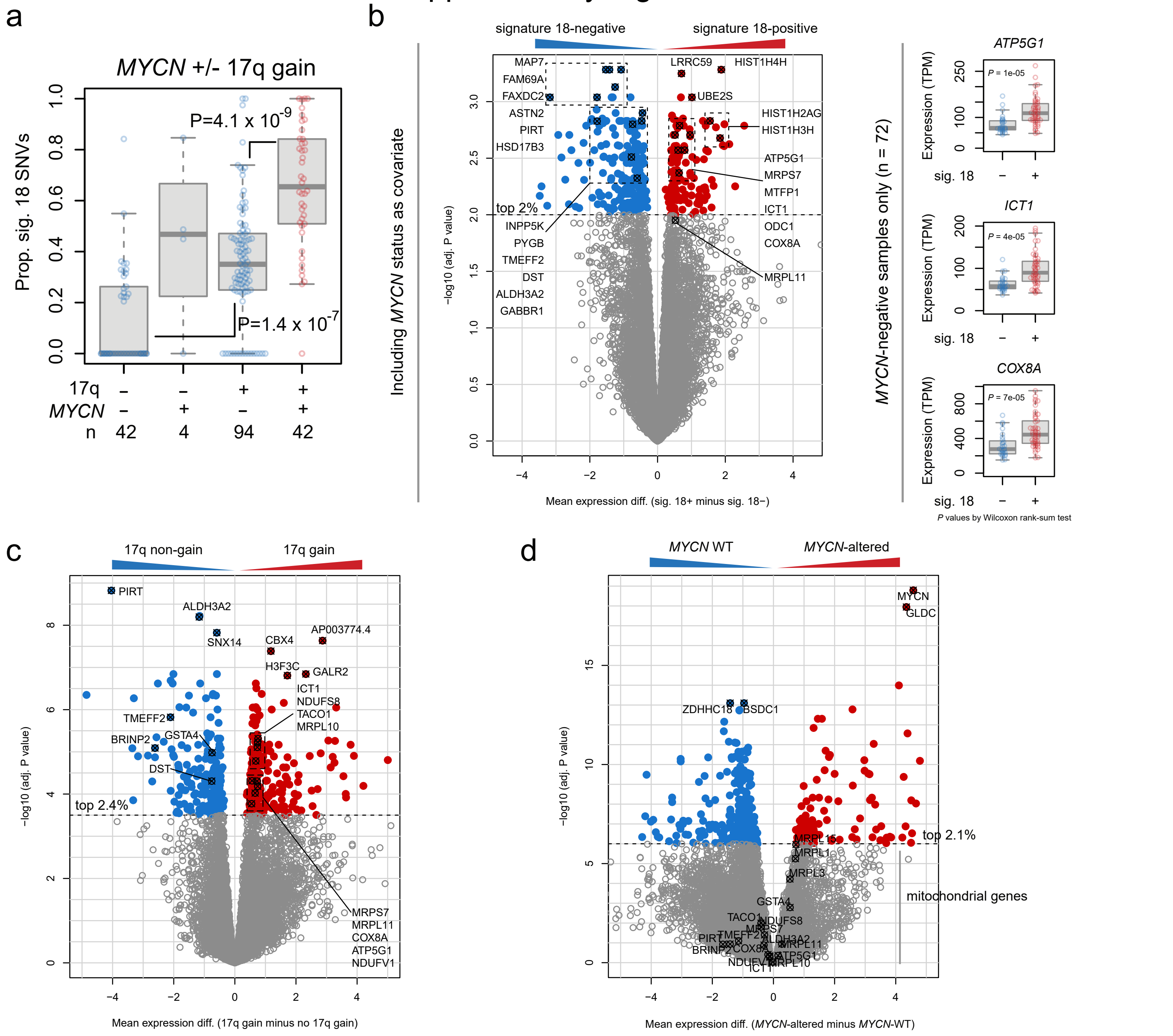

Supplementary Figure 19. Mitochondrial gene expression in signature 18-positive neuroblastomas is associated with 17q gains but not *MYCN*. (a) Boxplots showing proportion of SNVs caused by signature 18 in 182 diagnosis WGS samples, comparing samples with (+) or without (-) *MYCN* alterations and/or 17q gains. Box, interquartile range (25th to 75th percentile); middle bar, median. Whiskers are described in the R boxplot function documentation (a 1.5*interquartile range rule is used). The number of samples in each of the four groups is indicated at the bottom (n). *P* values are by two-sided Wilcoxon rank-sum test and are not shown for *MYCN*-only (without 17q gain) comparisons, since only four such samples were present. This shows that samples with 17q gains have increased signature 18 independent of *MYCN* status. (**b**) Left, differential gene expression analysis with Limma, comparing gene expression between signature 18-positive vs. -negative neuroblastomas, performed as in Fig. 4c from 88 diagnosis neuroblastomas with both WGS and RNA-Seq, except that *MYCN*-altered status was included as a covariate which removes the effects of *MYCN* from the differential expression analysis. This shows that most differentially expressed genes in Fig. 4c, including mitochondrial genes, were maintained as significant under these analytical conditions, and thus were not driven by *MYCN* alterations. The same sample numbers as Fig. 4c were included. Right shows boxplots comparing signature 18-negative vs. -positive samples, and y-axes indicate expression in TPM of indicated genes. Only *MYCN* non-altered samples are shown (n=72), including 45 signature 18-positive samples and 27 signature 18-negative samples. Box, median, and whiskers as in (a). (**c** and **d**) As in (b) except that 17q gains, in addition to *MYCN* status, were included as covariates. In this analysis, signature 18 was not significantly associated with expression of any gene independent of 17q gains and *MYCN*. (c) shows the genes correlating with 17q gains, showing that mitochondrial gene expression associated with signature 18 is closely linked to 17q gains, whereas mitochondrial gene expression is not due to *MYCN* gains (d). Source data are provided in Source Data file.

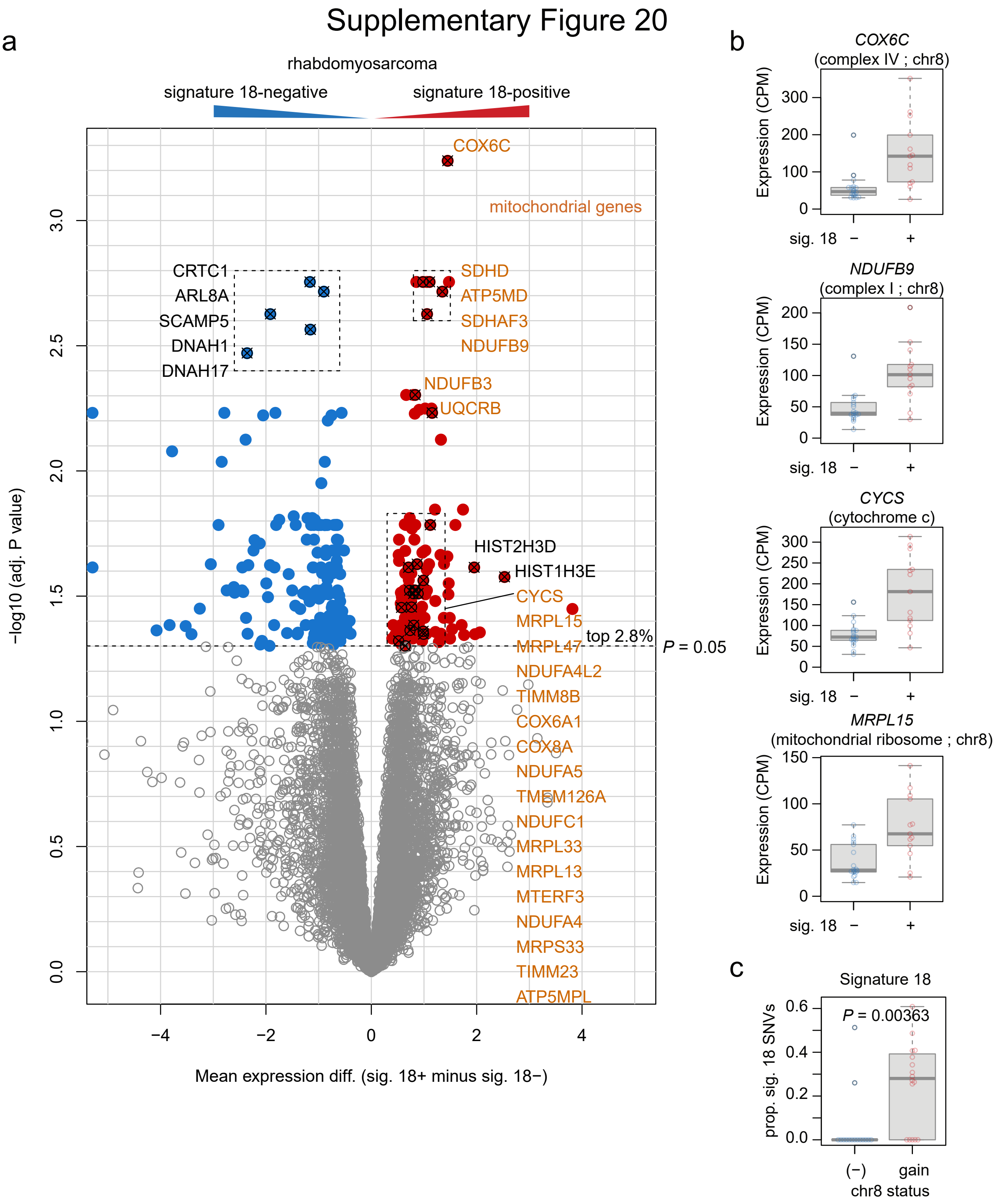

Supplementary Figure 20. Correlation between signature 18 and gene expression in rhabdomyosarcoma. (**a**) Volcano plot showing differential gene expression analysis performed with Limma on 31 rhabdomyosarcomas sequenced by WGS and RNA-Seq, split into signature 18-positive and -negative sample groups (n = 13 and n = 18, respectively). The figure is constructed similarly to Fig. 4c (please see legend for that figure for details), except that samples with adjusted *P* values of < 0.05 are highlighted in red (genes increased in signature 18-positive samples) or blue (genes increased in signature 18-negative samples). Genes with mitochondrial function are shown in orange text. (**b**) Expression of selected differentially expressed genes in signature 18-negative (n = 18) or -positive (n = 13) rhabdomyosarcomas, shown as boxplots as in Fig. 4d, with y-axis representing CPM. Box, interquartile range (25th to 75th percentile); middle bar, median. Whiskers are described in the R boxplot function documentation (a 1.5*interquartile range rule is used). (**c**) Proportion of signature 18 mutations in rhabdomyosarcomas without ((-), n = 15) or with (gain, n = 16) chromosome 8 copy gains. Box, interquartile range (25th to 75th percentile); middle bar, median. Whiskers are described in the R boxplot function documentation (a 1.5*interquartile range rule is used). *P* values are by two-sided Wilcoxon rank-sum test. Source data are provided as a Source Data file.

Supplementary Figure 21

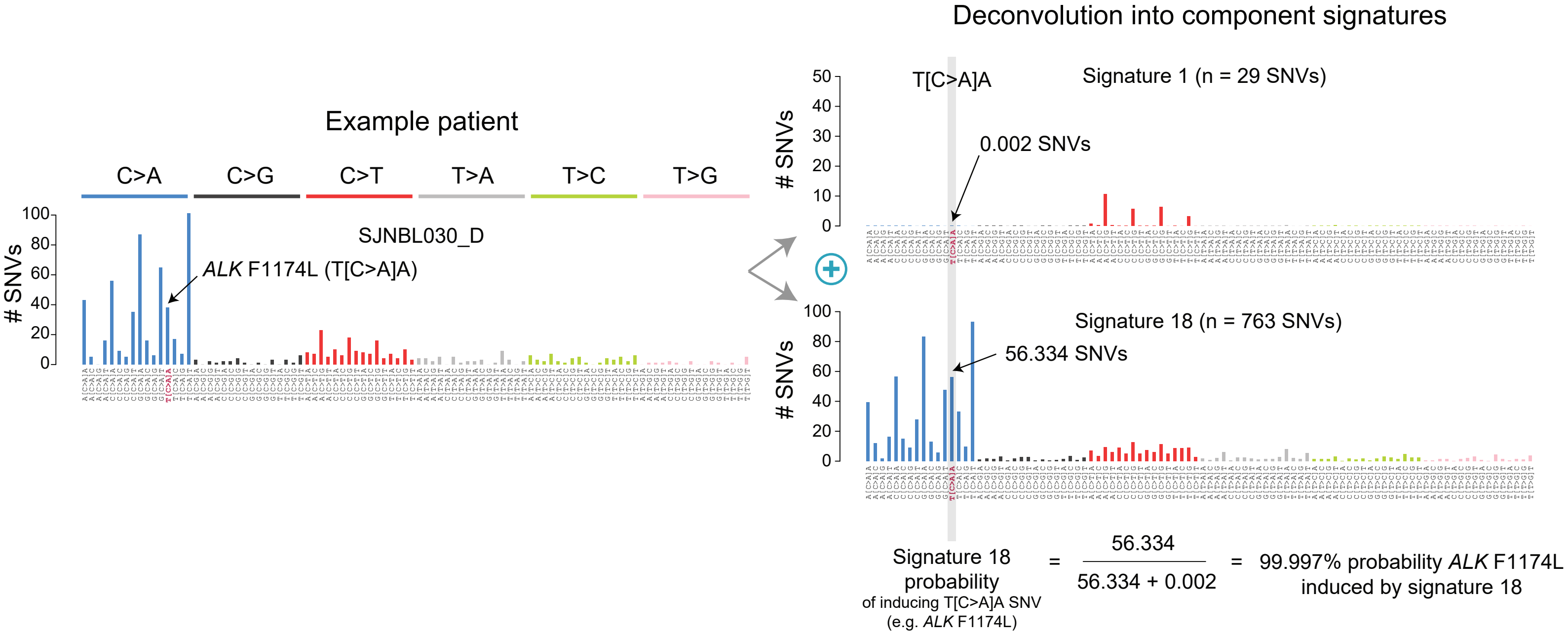

Supplementary Figure 21. Example of probability calculations showing that a specific mutation was induced by signature 18. Left shows the actual SNV trinucleotide context mutational profile if a neuroblastoma sample (SJNBL030_D). Each of the 96 classes of SNVs on the x-axis represents a specific mutation type (6 types shown at type) within a specific trinucleotide context. The patient had an *ALK* F1174L variant that was at the trinucleotide context T[C>A]A. Right shows the signature deconvolution of the sample, where a combination of two signatures (1 and 18) accurately reconstructed the samples profile with cosine similarity of 0.98. On the right panels, the number of mutations assigned to each signature (29 and 763, respectively) are multiplied by the published signature proportions at each trinucleotide context, yielding the probable number of mutations at each site caused by the signature (0.002 SNVs for signature 1, and 56.334 for signature 18). The probability that signature 18 caused the *ALK* F1174L variant (or any other variant in the sample at T[C>A]A) is thus ((56.334 + 0.002) / 56.334) = 0.99997, which makes sense given that signature 18 mutates at the context much more than signature 1. Source data are provided as a Source Data file.
