# Supplementary material for: Pan-neuroblastoma analysis reveals age- and signature-associated driver alterations
Source: Nat Commun. 2020 Oct 14;11:5183. doi: 10.1038/s41467-020-18987-4 (PMC7560655; doi:10.1038/s41467-020-18987-4)
Supplement: Supplementary file 3 — Description of Additional Supplementary Files [file 41467_2020_18987_MOESM3_ESM.pdf]

Description of Additional Supplementary Files

File Name: Supplementary Data 1
Description: **Cohort description, recurrent mutation distribution, and mutational signatures.** Each row represents one neuroblastoma sample with identifiers and aliases in columns A-C (note in particular the USI used by the Children's Oncology Group in C). Data source and diagnosis/relapse status are in columns D-E. Columns F-L indicate the sequencing platforms used and the repositories containing the raw genomic data. Clinical data are found in columns M-R. S indications coding-region mutation burden. Columns T-AQ indicate the presence or absence of indicated somatic alteration (yes indicates altered, no indicates not altered, NA indicates not known, such as when WGS is required to identify variants in the gene and only WES was performed). Mutational signature data are found in AR-AZ, and signature values represent number of SNVs caused by the signature (NA if WGS was not performed).

File Name: Supplementary Data 2
Description: **Gistic test result for copy number variations**. Direct output from Gistic showing significant copy number alterations. Each row represents a genomic region with significant copy gain or loss. Columns A-E indicate the peak name and region (GRCh37). Columns F-I indicate statistical values for each region.

File Name: Supplementary Data 3
Description: **Structural variation.** Each row represents a single structural variant detected in the sample indicated in column A. The first breakpoint's information is shown in columns B-F, and second breakpoint in columns G-K (GRCh37 coordinates). Read counts are in column L. Pathogenic status is in column M (B = benign, P = pathogenic, V = uncertain, L = likely). Driver gene(s) affected are in column N.

File Name: Supplementary Data 4
Description: Each row represents one coding-region SNV or indel detected in the sample in column A. Columns B-I indicate the variant's chromosomal location (GRCh37) and the gene annotation. Columns J-M show the number of mutant and total reads in the tumor and germline sample. Columns N-AA indicate additional information such as the data source, and statistical significance of recurrence based on GRIN or MutSigCV.

File Name: Supplementary Data 5
Description: **ALK N-terminal structural alterations.** The SV table shows the structural variants affecting the ALK N-terminal region in 5 patients, and the CNV table shows relevant copy number gains associated with these alterations (GRCh37 coordinates). LogRatio above zero indicates a copy gain.

File Name: Supplementary Data 6
Description: **Focal target CNV genes.** Each row represents a focal copy alteration detected from genomic sequencing of the sample in column A. Columns B-D indicate the genomic region affected (GRCh37 coordinates). Column E indicates the copy gain in $\log_2$ fold change from germline, such that values above zero indicate copy gain, and below zero indicate copy loss. Columns F-K indicate

additional information including the gene affected, whether it is a copy gain or loss, and the data source.
