# Supplementary material for: Pan-neuroblastoma analysis reveals age- and signature-associated driver alterations
Source: Nat Commun. 2020 Oct 14;11:5183. doi: 10.1038/s41467-020-18987-4 (PMC7560655; doi:10.1038/s41467-020-18987-4)
Supplement: Supplementary file 10 — Reporting Summary [file 41467_2020_18987_MOESM10_ESM.pdf]

nature research

Corresponding author(s): Jinghui Zhang, Michael A. Dyer, Michael D. Hogarty

Last updated by author(s): 2020-8-7

Reporting Summary

Nature Research wishes to improve the reproducibility of the work that we publish. This form provides structure for consistency and transparency in reporting. For further information on Nature Research policies, see Authors & Referees and the Editorial Policy Checklist .

Statistics

For all statistical analyses, confirm that the following items are present in the figure legend, table legend, main text, or Methods section.

n/a	Confirmed	
☐	☒	The exact sample size (*n*) for each experimental group/condition, given as a discrete number and unit of measurement
☒	☐	A statement on whether measurements were taken from distinct samples or whether the same sample was measured repeatedly
☐	☒	The statistical test(s) used AND whether they are one- or two-sided<br>*Only common tests should be described solely by name; describe more complex techniques in the Methods section.*
☐	☒	A description of all covariates tested
☐	☒	A description of any assumptions or corrections, such as tests of normality and adjustment for multiple comparisons
☐	☒	A full description of the statistical parameters including central tendency (e.g. means) or other basic estimates (e.g. regression coefficient) AND variation (e.g. standard deviation) or associated estimates of uncertainty (e.g. confidence intervals)
☐	☒	For null hypothesis testing, the test statistic (e.g. *F*, *t*, *r*) with confidence intervals, effect sizes, degrees of freedom and *P* value noted<br>*Give P values as exact values whenever suitable.*
☒	☐	For Bayesian analysis, information on the choice of priors and Markov chain Monte Carlo settings
☒	☐	For hierarchical and complex designs, identification of the appropriate level for tests and full reporting of outcomes
☒	☐	Estimates of effect sizes (e.g. Cohen's *d*, Pearson's *r*), indicating how they were calculated

Our web collection on statistics for biologists contains articles on many of the points above.

Software and code

Policy information about availability of computer code

Data collection	Standard Illumina instrumentation software (HiSeq2000 for most samples) and CGI software were used to collect the DNA and RNA sequencing data reported in this study.
Data analysis	We used BWA (version 0.5.9), CONSERTING (version 1.0), Bambino (version 1.6), CREST (version 1.0), RNApeg (version 1), GISTIC (version 2), Cis-X (version 1.4.0), CnvKit (version 0.9.1), SigProfiler (version 2.3.1), and SigProfilerSingleSample (version 1.3) for genomic analysis in this study. Most of the code used is publicly available and published as noted in the Methods section. RNApeg is available on Docker Hub under mnedmonson/public:rnapeg. Other custom Python and R code used was for routine data aggregation and plotting, and will be made available upon request.

For manuscripts utilizing custom algorithms or software that are central to the research but not yet described in published literature, software must be made available to editors/reviewers. We strongly encourage code deposition in a community repository (e.g. GitHub). See the Nature Research guidelines for submitting code & software for further information.

Data

Policy information about availability of data

All manuscripts must include a data availability statement. This statement should provide the following information, where applicable:

- Accession codes, unique identifiers, or web links for publicly available datasets
- A list of figures that have associated raw data
- A description of any restrictions on data availability

WGS data in the PCGP cohort can be obtained from EGA using accession EGAS00001000213 [https://www.ebi.ac.uk/ega/studies/EGAS00001000213]. WGS, WES, and RNA-seq data from TARGET can be accessed from dbGaP via accession phs000218 [https://www.ncbi.nlm.nih.gov/projects/gap/cgi-bin/study.cgi?study_id=phs000218.v22.p8]. COG WES bam files which are new to this study (n=317 samples) are available on EGA at accession EGAD00001005484 [https://www.ebi.ac.uk/ega/datasets/EGAD00001005484], where bam file names ending _D1.bam indicate tumor (diagnosis) samples and bam file names ending _G1.bam indicate germline samples. COG USI patient identifiers associated with each bam file, and the specific repository containing each patient’s raw data, can be found in Supplementary Data 1. All somatic alterations identified are recorded in Supplementary Data 1-6 and can also be viewed interactively using ProteinPaint at https://

pecan.stjude.cloud/proteinpaint/study/PanNeuroblastoma.Alterations. PCGP and Clinical Genomics bam files and somatic SNV data, including rhabdomyosarcoma data, are available via St. Jude Cloud [https://www.stjude.cloud/]. PCGP rhabdomyosarcoma data were published previously and are also available at EGAS00001000256 [https://www.ebi.ac.uk/ega/studies/EGAS00001000256]. Source data are provided with this paper in the accompanying Source Data file. Any remaining data are available within the Article, Supplementary files, or are available from the authors upon request.

Field-specific reporting

Please select the one below that is the best fit for your research. If you are not sure, read the appropriate sections before making your selection.

☒ Life sciences ☐ Behavioural & social sciences ☐ Ecological, evolutionary & environmental sciences

For a reference copy of the document with all sections, see nature.com/documents/nr-reporting-summary-flat.pdf

Life sciences study design

All studies must disclose on these points even when the disclosure is negative.

Sample size	702 samples' genomic data was aggregated from existing sources plus our 317 additional samples new to this study. No sample size calculation was performed as the purpose was to generate the largest possible cohort with readily available data and within financial and sample availability constraints. Samples were chosen for analysis based on availability. According to https://www.nature.com/articles/nature12912 (Lawrence et al.), sequencing of ~700 samples should allow discovery of some 3-10 new recurrent driver genes.
Data exclusions	Samples with pathologically determined low tumor purity were excluded. The pre-established criteria for inclusion were >=60% purity for the 317 COG samples new to this study, or >=75% purity for the TARGET samples. Library preparation failures were also excluded.
Replication	We did not verify reproducibility as our goal was to aggregate previously published and newly sequenced neuroblastoma genomic data, in order to create a large neuroblastoma genomic dataset.
Randomization	Patients were not allocated into experimental groups. This was a retrospective, descriptive study of the genomic basis of neuroblastoma.
Blinding	Patients were not allocated into experimental groups, so no blinding was performed. This was a retrospective, descriptive study of the genomic basis of neuroblastoma.

Reporting for specific materials, systems and methods

We require information from authors about some types of materials, experimental systems and methods used in many studies. Here, indicate whether each material, system or method listed is relevant to your study. If you are not sure if a list item applies to your research, read the appropriate section before selecting a response.

Materials & experimental systems

n/a	Involved in the study	
☒	☐	Antibodies
☒	☐	Eukaryotic cell lines
☒	☐	Palaeontology
☒	☐	Animals and other organisms
☐	☒	Human research participants
☒	☐	Clinical data

Methods

n/a	Involved in the study	
☒	☐	ChIP-seq
☒	☐	Flow cytometry
☒	☐	MRI-based neuroimaging

Human research participants

Policy information about studies involving human research participants

Population characteristics	702 pediatric neuroblastoma samples were analyzed and divided into 3 age groups (0-1.5 years, 1.5-5 years, and 5+ years), with median age of 2.7 years. The cohort is approximately 60% male, consistent with known higher frequency of neuroblastoma in males. 679 samples are from diagnosis and 23 from relapsed disease. The dataset features the breadth of neuroblastoma risk groups. 31 rhabdomyosarcoma samples were also included from the PCGP and from patients enrolled in the St. Jude Clinical Genomics program.
Recruitment	Participants were recruited to the study primarily from Children's Oncology Group member institutions (TARGET and COG datasets), or at Memorial Sloan-Kettering Cancer Center (PCGP dataset). The PCGP dataset and much of the TARGET dataset, particularly the exome samples, are focused on high-risk disease, which may identify genomic aberrations enriched in high-risk disease. By contrast, the COG cohort is focused on low-risk disease, which may identify fewer genomic aberrations or aberrations enriched in low-risk disease. Combining these datasets enables us to understand the breadth of neuroblastoma genetics. In several of our analyses, we controlled for risk status by analyzing only high-risk (stage 4) patients, in addition to our cohort-wide analysis. 31 rhabdomyosarcoma samples from St. Jude were also included from the PCGP and from patients enrolled in the St. Jude Clinical Genomics program.
Ethics oversight	The Children's Hospital of Philadelphia, Memorial Sloan Kettering Cancer Center, St. Jude Children's Research Hospital.

Note that full information on the approval of the study protocol must also be provided in the manuscript.
